# Time Delay and Long-Range Connection Induced Synchronization Transitions in Newman-Watts Small-World Neuronal Networks

**DOI:** 10.1371/journal.pone.0096415

**Published:** 2014-05-08

**Authors:** Yu Qian

**Affiliations:** 1 Nonlinear Research Institute, Baoji University of Arts and Sciences, Baoji, China; 2 Center for Systems Biology, Soochow University, Suzhou, China; 3 State Key Laboratory of Theoretical Physics, Institute of Theoretical Physics, Chinese Academy of Sciences, Beijing, China; University of Maribor, Slovenia

## Abstract

The synchronization transitions in Newman-Watts small-world neuronal networks (SWNNs) induced by time delay 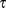 and long-range connection (LRC) probability 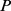 have been investigated by synchronization parameter and space-time plots. Four distinct parameter regions, that is, asynchronous region, transition region, synchronous region, and oscillatory region have been discovered at certain LRC probability 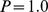 as time delay is increased. Interestingly, desynchronization is observed in oscillatory region. More importantly, we consider the spatiotemporal patterns obtained in delayed Newman-Watts SWNNs are the competition results between long-range drivings (LRDs) and neighboring interactions. In addition, for moderate time delay, the synchronization of neuronal network can be enhanced remarkably by increasing LRC probability. Furthermore, lag synchronization has been found between weak synchronization and complete synchronization as LRC probability 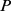 is a little less than 1.0. Finally, the two necessary conditions, moderate time delay and large numbers of LRCs, are exposed explicitly for synchronization in delayed Newman-Watts SWNNs.

## Introduction

Synchronization phenomena are common in nature and can be extensive observed in various realistic systems, especially in neuronal networks, biological systems and ecological systems [1], [2]. Synchronization has been widely studied both theoretically and experimentally for decades. Several kinds of synchronization have been discovered in theoretical researches, such as complete synchronization, weak synchronization, lag synchronization, phase synchronization and generalized synchronization [3]–[7]. Complete synchronization indicates the coincidence of states of coupling systems, 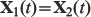 [3]. Lag synchronization described in Ref. [5] means the coincidence of shifted in time states of two systems, 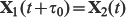. Experimental studies have shown that synchronous oscillations can emerge in many special areas of brain, especially in olfactory system or hippocampal region [8]–[10]. In recent years, synchronization in neuronal networks and brain systems has attracted much attention. Synchronous oscillations in these systems are related to some specific and important physiological functions, such as olfaction [11], visual perception [12], cognitive processes [13], and information processing [14].

Recently “small-world” network has been proposed by Watts and Strogatz, which takes into account both local and long-range interactions [15]. It is found that the existence of a small fraction of long-range connections (LRCs) can essentially change the features of the given systems [16]–[19]. These LRCs do exist in neuronal networks and do play crucial roles in deciding the specific physiological functions. The interactions from the long-range connected neurons must be time delayed due to the finite propagation velocities in the conduction of signals along neuron axons [20]. And the effects of time delays on self-organized spatiotemporal dynamics in neuronal systems have been extensively investigated. Lots of interesting phenomena have been discovered in recent decades [21]–[36]. For example, Dhamala et al. have investigated the enhancement of neural synchrony by time delay [21]. Ko et al have found that time delay can destabilize synchronous states and induce near-regular wave states [23]. Significantly, Wang et al. have discovered that time delays can enhance the coherence of spiral waves [27], tame desynchronized bursting [28], induce stochastic resonances [29] and synchronization transitions [30]–[32], and can cause synchronous bursts [33] and complex synchronous behavior [34]. Moreover, Yu et al. have demonstrated the synchronization transitions in delayed neuronal networks with hybrid synapses [35], [36]. Although remarkable advances have been achieved in the field of delayed neuronal networks, the underlying mechanisms behind time delay induced spatiotemporal dynamic and related synchronization transitions are far from being fully understood. In addition, the lag synchronization, to our knowledge, has not been identified in delayed neuronal network. These are the tasks we aim to explore.

In this paper we extend the subject by systematically investigating time delay and long-range connection induced synchronization transitions in Newman-Watts small-world neuronal networks (SWNNs). By introducing synchronization parameter and plotting spatiotemporal patterns, four distinct parameter regions, i.e., asynchronous region, transition region, synchronous region and oscillatory region, have been found at certain LRC probability 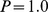. Interestingly, desynchronization and oscillating behaviour of the order parameter are observed in oscillatory region. More importantly, the mechanisms of synchronous oscillations and the transition from non-synchronization to complete synchronization are discussed. Moreover, we consider the spatiotemporal patterns obtained in delayed Newman-Watts SWNNs are the competition results between long-range drivings (LRDs) and neighboring interactions. A new order parameter, LRD proportion, is used to verify our point of view. And the four distinct parameter regions can also be revealed by LRD proportion clearly. In addition, for moderate time delay, the synchronization of neuronal network can be enhanced remarkably by increasing LRC probability. Furthermore, lag synchronization has been found between weak synchronization and complete synchronization as LRC probability 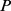 is a little less than 1.0. And the mechanism is revealed. Finally, the two necessary conditions, moderate time delay and large numbers of LRCs, are exposed explicitly for synchronization in delayed Newman-Watts SWNNs.

## Mathematical Model and Setup

We start from a one-dimensional (1D) regular ring that comprises 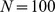 identical excitable 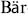-Eiswirth neurons[37] with periodic boundary condition, and each neuron has two nearest neighbors. The evolvement of the 1D neuronal network is governed by the following equations:



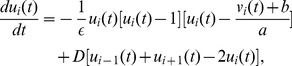
(1)

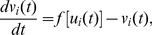
(2)where 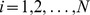. The function 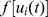 takes the form: 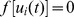 for 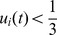; 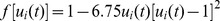 for 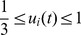; and 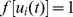 for 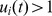. Here variables 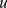 and 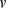 are the activator and inhibitor variables, respectively. The small relaxation parameter 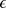 represents the time ratio between activator 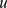 and inhibitor 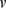. The dimensionless parameters 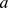 and 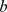 denote the activator kinetics with 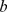 effectively controlling the excitation threshold. 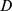 is the coupling intensity which decides the interaction strength between neighboring neurons. The system parameters are kept throughout this paper as 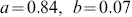, 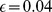 and 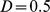. Therefore, the local dynamics can describe typical excitability of neurons where 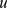 represents the membrane potential, 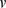 is the somatic inhibitory current. The diffusive couplings simulates electrical conjunction interaction between neurons.

Based on the 1D periodic regular ring, we construct delayed Newman-Watts SWNNs [38] by introducing LRCs such that each neuron receives an unidirectional time delayed LRD from a randomly chosen cell with probability 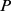 [39], [40]. We thus add an additional coupling term to Eq. (1) if neuron 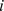 receives an unidirectionally time delayed LRD from cell 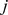. Now the delayed Newman-Watts SWNN is governed by the following equations:
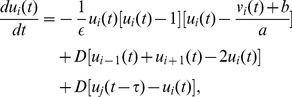
(3)

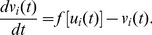
(4)


**Figure 1:**
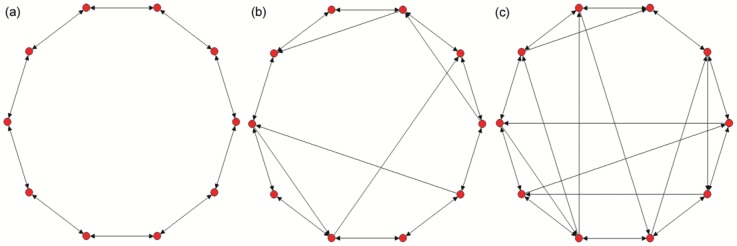
Schematic diagram of the considered Newman-Watts small-world neuronal networks for different long-range connection (LRC) probability 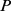 with 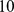 neurons. (a) 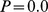 (one-dimensional regular ring with periodic boundary condition); (b) 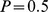; (c) 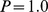. Here we should mention that the interactions between neighboring neurons are bidirectional (shown by bidirectional arrowed lines), while the long-range drivings (LRDs) are unidirectional (shown by unidirectional arrowed lines). Time delays are only considered in these unidirectional LRDs.

In Eq. (3) cell 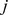 is randomly chosen in the 1D periodic regular ring and 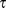 is the time delay in information transmission. By manipulating LRC probability 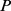, we can obtain different kinds of time delayed Newman-Watts SWNNs. The schematic diagram of the considered networks for different LRC probability with 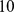 neurons is illustrated in Fig. 1. Here we should mention that for a given LRC probability there are a lot of network realizations. For a specific network structure, the interactions between neighboring neurons are bidirectional (shown by bidirectional arrowed lines), while the LRDs are unidirectional (shown by unidirectional arrowed lines). Time delays are only considered in these unidirectional LRDs, which will cause inhomogeneity in information transmission between neighboring and long-range interactions. As we know that the interactions from neighboring neurons are usually instantaneous in actual biological systems. And the LRDs from distant cells will have time delays due to the finite propagation velocities. Therefore, the model considered in present paper may be more realistic, and the results obtained may be more practical.

In this paper, the delayed Newman-Watts SWNNs are integrated by forward Euler integration scheme with time step 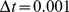. The initial variables (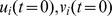) are randomly given between 0 and 1 for each simulation. To investigate the synchronization transitions in delayed Newman-Watts SWNNs quantitatively, the synchronization parameter 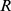 will be used, which has been introduced in the previous study [41]. It is numerically calculated as:
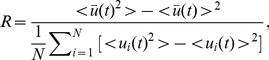
(5)where



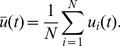
(6)The angular brackets denote the average over time. In present paper the synchronization parameters are calculated over last 30 time units. From Eq. (5) it is evident that the larger the synchronization parameter 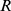 is, the more synchronization is realized in neuronal network. Accordingly, the value of 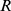 close to unity indicates all neurons in the network are in complete synchronization. Therefore, the synchronization parameter 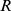 is an excellent indicator to reveal the spatiotemporal synchronization in delayed Newman-Watts SWNNs and the related transitions.

To guarantee the statistical accuracy with respect to the network structure and initial condition, 10 independent samples are executed for each set of parameter values in the simulation. And we will use
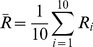
(7)as an order parameter to measure the degree of synchronization and the related transitions induced by time delay and long-range connection in Newman-Watts SWNNs.

## Results

### Time Delay Induced Synchronization Transitions

**Figure 2:**
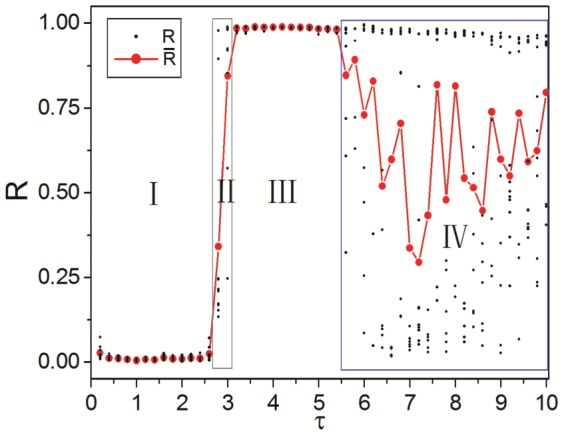
Time delay induced synchronization transitions. Dependence of synchronization parameters 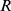 (10 samples for each 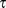, depicted by black dots) and 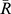 (the average of 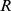
s for 10 samples, depicted by red dots) on time delay 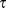 at 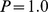. Four distinct parameter regions, i.e., asynchronous region (domain I for small 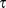), transition region (domain II for narrow region of time delay 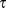, indicated by grey rectangle), synchronous region (domain III for moderate 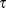) and oscillatory region (domain IV for large 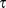, indicated by blue rectangle) are revealed.

**Figure 3:**
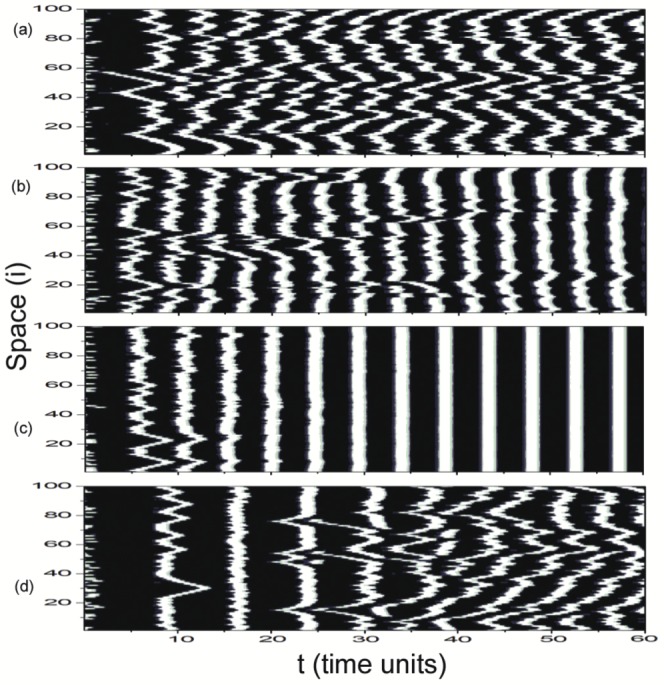
Space-time plots of 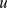 for different time delay 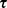 at 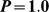. (a) 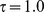 (asynchronous state), (b) 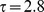 (weak synchronization), (c) 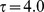 (complete synchronization), (d) 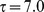 (desynchronized state). The figures are plotted in greyscale from black (lowest value at 0.0) to white (highest value at 1.0). And this greyscale will be used throughout this paper.

In this part, we firstly investigate time delay induced synchronization transitions in Newman-Watts SWNNs at certain LRC probability. Figure 2 displays the dependence of synchronization parameters 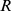 (10 samples for each 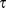, depicted by black dots) and 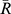 (the average of *R*s for 10 samples, depicted by red dots) on time delay 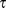 at 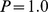. Four distinct parameter regions have been revealed by synchronization parameters as time delay is increased. When time delay is small (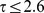), synchronization parameters are all close to zero. It indicates that the states of individual neurons are significantly different and the whole network oscillates asynchronously at all (domain I in Fig. 2, called as asynchronous region). It means that small time delay has no effect on synchronization in delayed Newman-Watts SWNNs. A typical asynchronous spatiotemporal pattern is shown in Fig. 3(a) for 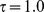. In the white regions, the nodes fire, while in the black ones they are quiescent. Time passes from left to right. Most of neurons in the network oscillate asynchronously and irregular spatiotemporal dynamics is observed. As 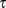 is in the narrow region of 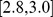, some synchronization parameters increase abruptly. It indicates that synchronous performance of neuronal network improves remarkably in some samples. A weak synchronization state for 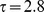 is revealed in Fig. 3(b). The excitatory fronts are more ordered both in time and space. Time delay induced synchronization transition has been detected in Newman-Watts SWNNs. And we call this narrow parameter region as the transition region (domain II in Fig. 2, indicated by grey rectangle).

When time delay is moderate (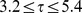, domain III in Fig. 2), synchronization parameters 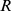 and 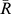 jump to unity simultaneously. It implies that moderate time delay in information transmission can induce complete synchronization in Newman-Watts SWNNs. Therefore, synchronous region is defined in this parameter region. Fig. 3(c) exhibits a completely synchronous spatiotemporal pattern for 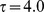. All neurons in the network fire simultaneously and damp to their rest state together. As time delay is further increased (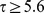), to our surprise, desynchronization occurs in Newman-Watts SWNNs. A distinct new parameter region, composed by asynchronous state, weak synchronization and complete synchronization, has been discovered. And oscillating behaviour of the order parameter is detected. Accordingly, we call this parameter region as the oscillatory region (domain IV in Fig. 2, indicated by blue rectangle). Fig. 3(d) displays a typical desynchronized spatiotemporal dynamics in oscillatory region at 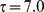. Large time delay can effectively improve synchronization in the beginning (can be indicated by the second excitatory front in Fig. 3(d)). However, the ordered excitatory front degenerates and desynchronization occurs as the system evolves. Finally, asynchronous state is obtained in oscillatory region. According to the results shown in Fig. 2, we can conclude that moderate time delay is needed for synchronization in delayed Newman-Watts SWNNs.

**Figure 4:**
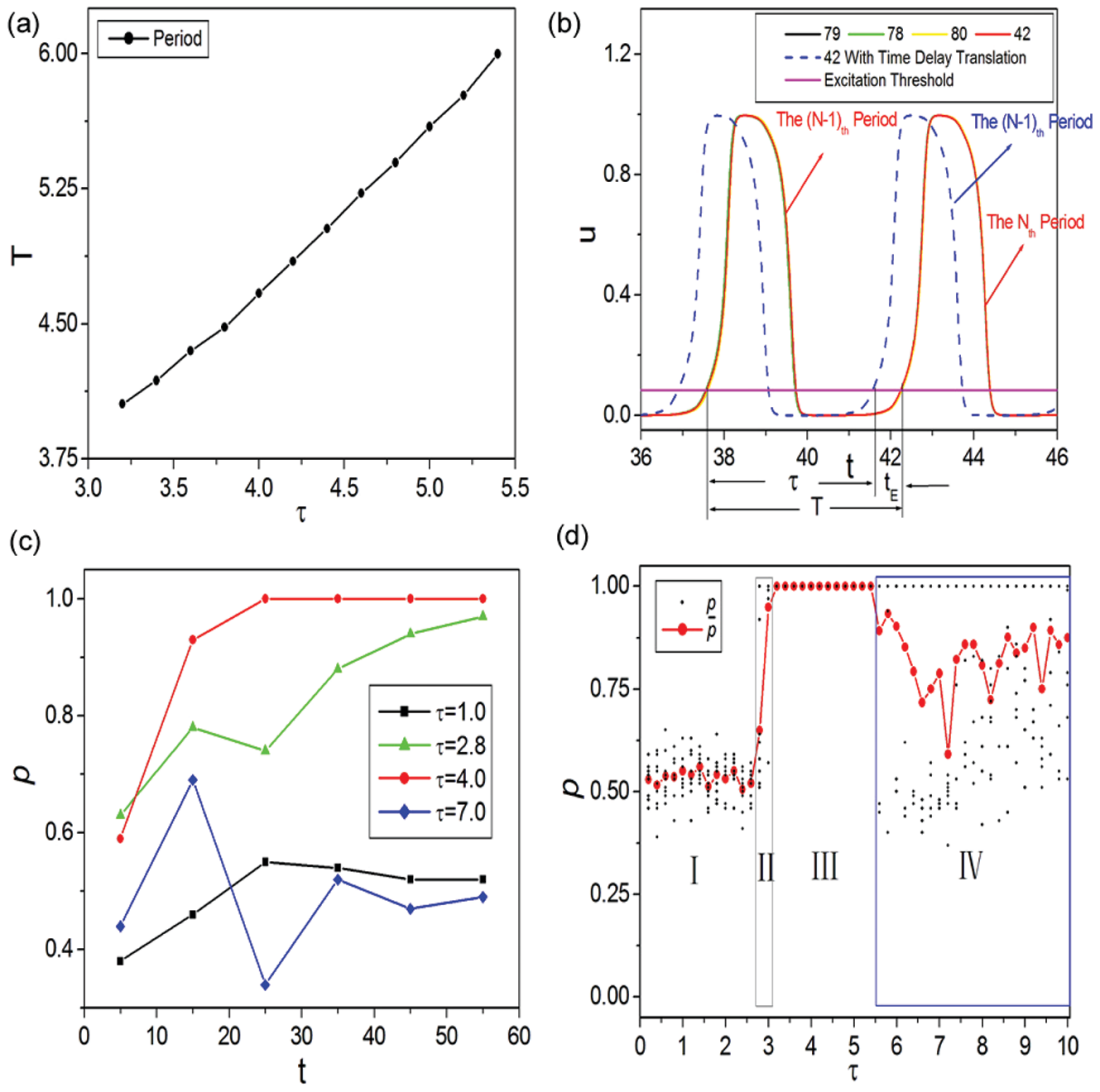
Dynamical analysis of synchronous oscillations and time delay induced synchronization transitions. (a) Dependence of oscillation period 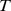 on time delay 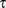 in synchronous region. (b) Time series 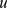 of neurons 79 (shown by black curve), 78 and 80 (two neighboring neurons of 79, shown by green and yellow curves) and 42 (the LRD neuron of 79, shown by red curve) of Fig. 3(c). The blue dashed curve denotes time series 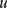 of neuron 42 with time delay translation. The pink line indicates excitation threshold. The oscillation period 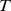 is composed by time delay 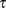 and excitation time 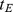. (c) The LRD proportion 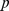 between adjacent intervals for different time delay 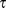 (corresponding to Figs. 3(a)–(d)). (d) Dependence of LRD proportion 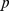 (10 samples for each 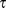, depicted by black dots) and 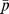 (the average of 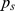 for 10 samples, depicted by red dots) on time delay 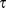. The four distinct parameter regions can also be revealed by LRD proportion clearly.

For further investigating the synchronous oscillations, the dependence of oscillation period 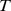 on time delay 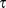 in synchronous region is shown in Fig. 4(a). It is seen that synchronization oscillation period is monotonously increased with time delay. And approximate linear relationship is revealed. However, a time difference between 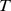 and 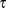 can be detected. To explain the above phenomenon, time series 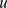 of neurons 79 (shown by black curve), 78 and 80 (two neighboring neurons of 79, shown by green and yellow curves) and 42 (the LRD neuron of 79, shown by red curve) of Fig. 3(c) are shown in Fig. 4(b). The blue dashed curve denotes time series 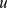 of neuron 42 with time delay translation. The pink line indicates excitation threshold. From Fig. 4(b) we can find that synchronization oscillation period 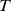 is composed by time delay 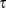 and excitation time 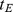. That's why there exists a time difference between synchronization oscillation period and time delay.

The mechanism of synchronous oscillations can also be explained by Fig. 4(b). As complete synchronization is achieved in delayed Newman-Watts SWNNs, all neurons can excite simultaneously and damp to their rest state together, oscillate just as a single cell (can be indicated by the overlap of the four solid curves). Since time delays exist in LRCs, neurons can be excited synchronously again by their corresponding delayed LRDs (can be indicated by the black solid and blue dashed curves). Synchronous oscillations can self-sustain in delayed Newman-Watts SWNNs in this manner (such as the two excitation periods shown in Fig. 4(b)). However, due to the existence of refractory period for excitable neuron, a minimal time delay 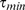 is needed for LRDs sustaining synchronous oscillations. Accordingly, complete synchronization can emerge in delayed Newman-Watts SWNNs as 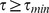. Based on the results shown in Fig. 2, we can find 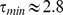 under current parameter settings. Now the transition from non-synchronization to complete synchronization can be explained as follow: For small time delays (i.e., 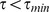), LRDs can not occupy the whole network entirely and simultaneously due to the existence of refractory period for excitable dynamics. Neurons in the network are mostly driven by their neighbors. As a result, zigzag excitation fronts (i.e., asynchronous spatiotemporal patterns) are obtained. As 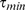 is reached, LRDs can dominate the neuronal network absolutely, and complete synchronization can emerge in delayed Newman-Watts SWNNs.

From the above discussion, we can find that LRDs play a key role in synchronization transition. To qualitatively investigate the effects of LRDs on the spatiotemporal dynamics obtained in delayed Newman-Watts SWNNs, the LRD proportion 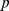 is used, which can be calculated as:
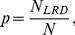
(8)where 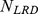 is the total number of neurons driven by LRDs. The evolvement of LRD proportion 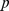 between adjacent intervals for different time delay 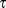 (corresponding to Figs. 3(a)–d)) is shown in Fig. 4(c). As time delay is small (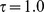, below 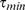, shown by black squares), 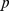 increases slightly at first and then tends to 0.5. It indicates that the neuronal network is governed by local and long-range drivings together. Accordingly, irregular asynchronous spatiotemporal dynamics of Fig. 3(a) is obtained. When 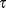 is in the transition region (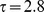, close to 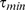, shown by green triangles), LRD proportion 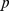 increases abruptly, but can never reach 1.0. It means that most of neurons in the network are sustained by LRDs, and can fire simultaneously. However, few neurons are still excited by their corresponding neighbors. Therefore, weak synchronization can be observed. As synchronous region is reached (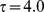, beyond 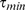, shown by red dots), LRD proportion 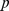 jumps to unity rapidly. With the help of moderate time delay, LRDs can suppress neighboring interactions to dominate the system entirely. All neurons in the network can be excited by their corresponding LRDs simultaneously, and complete synchronization can emerge in delayed Newman-Watts SWNNs. When time delay is large (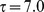, also beyond 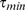, shown by blue diamonds), 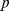 increases abruptly at first and goes through a peak, then deceases monotonously, and finally tends to 0.5. It means that LRDs can take effect so long as 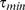 is reached. And LRDs can dominate the neuronal network in the beginning and weak synchronization such as the second excitatory front of Fig. 3(d) can be achieved. However, LRD loses its predominance as the system evolves. It may be caused by the too long resting time which can increase the chance for neighboring interactions. As a result, the ordered excitatory front degenerates and desynchronization occurs in the oscillatory region. So we consider that too large time delay may be harmful for synchronization to a certain degree.

Based on the above discussion, we can infer that the mechanism behind spatiotemporal dynamics obtained in delayed Newman-Watts SWNNs is the competition between LRDs and neighboring interactions. This kind of competition is caused by inhomogeneity in information transmission between neighboring and long-range interactions of the present model. More importantly, the competition results, which will decide the spatiotemporal dynamics in the network, are largely dependent on time delays. Therefore, we can expect that the LRD proportion is also a good indicator to study the synchronization transitions in delayed Newman-Watts SWNNs. The dependence of LRD proportion 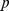 (10 samples for each 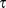, depicted by black dots) and 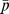 (the average of 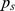 for 10 samples, depicted by red dots) on time delay 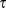 is shown in Fig. 4(d). The four distinct parameter regions are revealed by LRD proportion clearly. Moreover, we can also find that moderate time delay can help LRDs to beat neighboring interactions to dominate the network absolutely. The conclusion that moderate time delay is needed for synchronization in delayed Newman-Watts SWNNs is further verified.

### LRC Induced Synchronization Transitions

**Figure 5:**
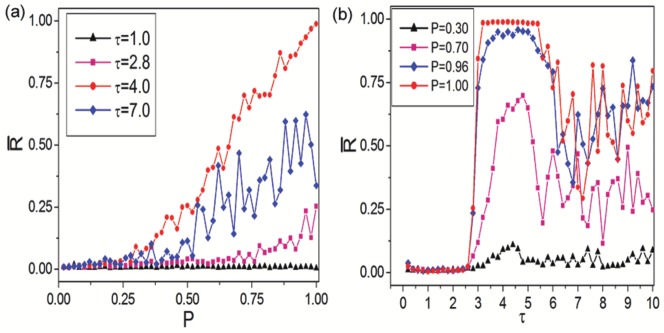
LRC induced synchronization transitions. (a) Dependence of synchronization parameter 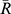 on LRC probability 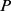 for different time delay 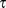. (b) Dependence of synchronization parameter 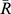 on time delay 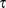 for different LRC probability 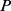.

From the above understanding we can find that LRDs play an important role in deciding the spatiotemporal dynamics. Therefore, a detailed study on LRC induced synchronization transitions needs to be taken in delayed Newman-Watts SWNNs. Fig. 5(a) displays the dependence of synchronization parameter 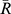 on LRC probability 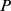 for different time delay 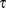. For small time delay (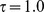, below 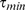, shown by black triangles), LRDs can not occupy the system due to the existence of refractory period. As a result, LRCs have no effect on synchronization transitions in asynchronous region. When time delay is in transition region (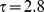, close to 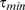, shown by pink squares), few LRDs can occupy the neuronal network under this circumstance. Therefore, lots of LRCs are needed to slightly improve the synchronization. For moderate time delay (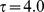, beyond 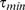, shown by red dots), LRDs can suppress neighboring interactions to dominate the system entirely. Consequentially, synchronization in delayed Newman-Watts SWNNs can be enhanced remarkably by increasing LRC probability 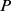. For large time delay (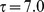, also beyond 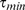, shown by blue diamonds), synchronization of delayed Newman-Watts SWNN improves as LRC probability increases. However, as we have identified, too large time delay can increase the chance for neighboring interactions and is harmful for synchronization to a certain degree, oscillating behaviour of the order parameter can be observed. Fig. 5(b) displays the dependence of synchronization parameter 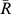 on time delay 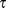 for different LRC probability 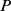. An optimal time delay interval is needed to enhance the synchronization for Newman-Watts SWNNs. The centers of optimal time delay interval are all around 4.5 and are largely independent of LRC probability. The width of optimal time delay interval broadens as LRC probability increases.

**Figure 6:**
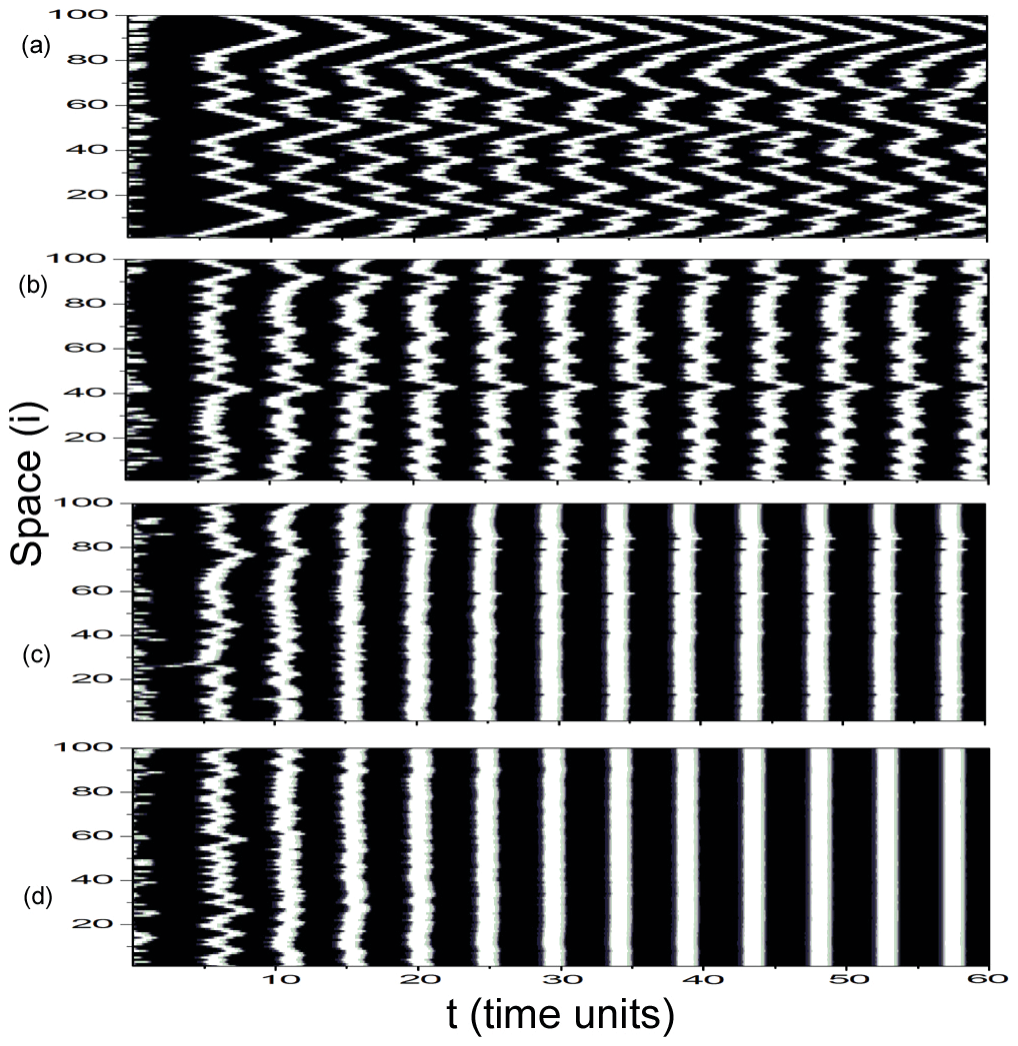
Space-time plots of 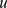 for different LRC probability 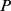 at 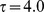. (a) 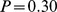 (asynchronous state), (b) 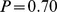 (weak synchronization), (c) 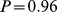 (lag synchronization), (d) 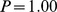 (complete synchronization).

To give more intuitive understanding on LRC induced synchronization transitions in delayed Newman-Watts SWNNs, space-time plots of 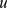 for different LRC probability 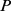 at 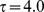 is given in Fig. 6. Remarkable enhancement of synchronization induced by LRCs in delayed Newman-Watts SWNNs is revealed obviously. Besides the asynchronous state (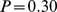 for Fig. 6(a)), weak synchronization (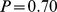 for Fig. 6(b)) and complete synchronization (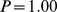 for Fig. 6(d)), another new synchronization mode has been found at 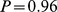 and is shown in Fig. 6(c). From visual assessment, we guess this kind of new synchronization mode is the lag synchronization. To test our idea, the similarity function is introduced, which was proposed to detect lag synchronization [5]. It is numerically calculated as:
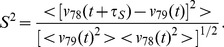
(9)


**Figure 7:**
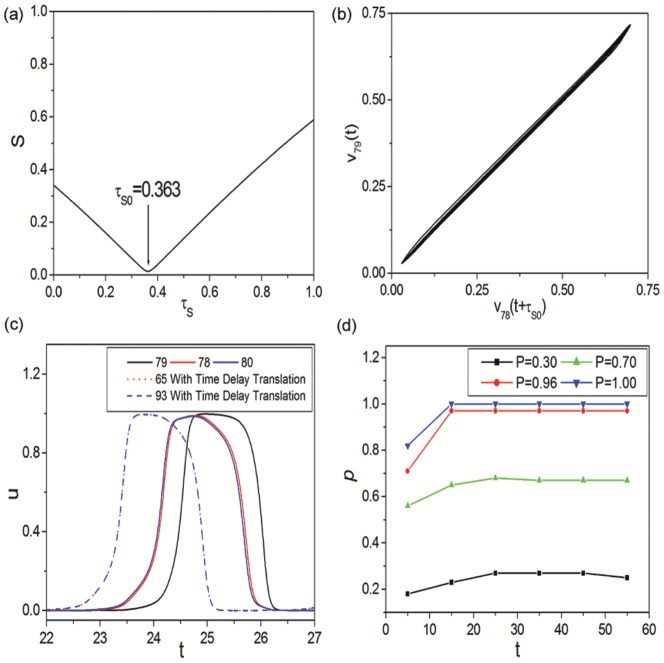
Dynamical analysis of lag synchronization and LRC induced synchronization transitions. (a) Dependence of similarity function 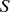 on time shift 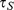. The minimal value of 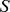 appears at 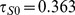, which indicates the lag synchronization between neurons 79 and 78 of Fig. 6(c). (b) Projection of the attractor on the time shifted plane (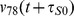, 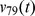). It demonstrates that the state of neuron 79 is delayed in time with respect to neuron 78. (c) Time series 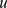 of neurons 79 (without LRC, shown by black curve), 78 and 80 (two neighboring neurons of 79, shown by red and blue curves). The red dotted and blue dashed curves denote time series 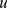 of neurons 65 and 93 (the two LRD neurons of 78 and 80) with time delay translation, respectively. Lag synchronization is discovered in delayed Newman-Watts SWNN and the mechanism is also revealed. (d) The LRD proportion 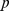 between adjacent intervals for different LRC probability 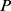 (corresponding to Figs 6(a)–(d)).

Here 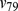 and 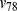 are the time series 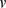 of neurons 79 and 78 of Fig. 6(c). And 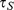 is the time shift. Fig. 7(a) displays the dependence of similarity function 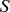 on time shift 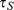. The minimal value of 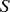 appears at 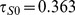, which indicates the lag synchronization between neurons 79 and 78. Fig. 7(b) shows the projection of the attractor on the time shifted plane (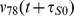, 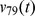). It demonstrates that the state of neuron 79 is delayed in time with respect to neuron 78. Accordingly, lag synchronization has been confirmed in delayed Newman-Watts SWNN. To explain the mechanism of lag synchronization, time series 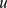 of neurons 79 (without LRC, shown by black curve), 78 and 80 (two neighboring neurons of 79, shown by red and blue curves) of Fig. 6(c) are shown in Fig. 7(c). And the red dotted and blue dashed curves denote time series 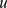 of neurons 65 and 93 (the two LRD neurons of 78 and 80) with time delay translation, respectively. As LRC probability 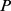 is a little less than 1.0, some neurons in network will have no LRCs due to finite connection probability. All neurons without LRCs must be driven by their neighbors. And these neighboring neurons are excited by their corresponding delayed LRDs. The successive driving relationship is revealed in Fig. 7(c). And lag synchronization between neurons without LRCs and their corresponding neighbors is identified. Therefore, we can observe lag synchronization in delayed Newman-Watts SWNNs as LRC probability 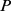 is a little less than 1.0 at moderate time delay. Fig. 7(d) exhibits the LRD proportion 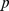 between adjacent intervals for different LRC probability 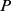 at 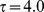 (corresponding to Figs. 6(a)–(d)). Anticipated LRD proportions can be quickly approached so long as time delay is moderate. And large numbers of LRCs are needed to dominate the network for synchronization under this circumstance.

According to the results obtained in this part, the conclusion that moderate time delay can help LRDs to dominate the network has been verified again. And large numbers of LRCs are needed for synchronization under this circumstance. Therefore, the two necessary conditions, moderate time delay and large numbers of LRCs, are exposed explicitly for synchronization in delayed Newman-Watts SWNNs.

### The Combined Effects on Synchronization Transitions

**Figure 8:**
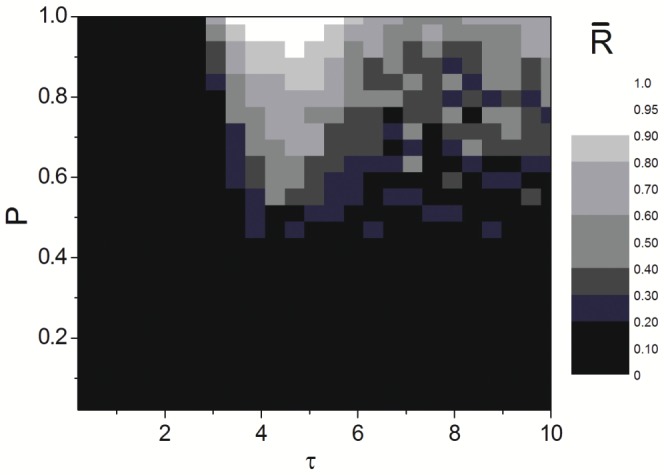
The combined effects on synchronization transitions. Dependence of synchronization parameter 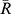 on time delay 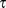 and LRC probability 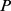.

To have a overall inspection of time delay and LRC induced synchronization transitions in Newman-Watts SWNNs, the contour plot of synchronization parameter 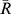 in the plane 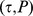 is revealed in Fig 8. The color intensity denotes the synchronization degree in delayed Newman-Watts SWNNs. Specifically, lighter color representing larger synchronization parameter, which indicates higher degree of synchronization. The four distinct parameter regions, i.e., asynchronous region, transition region, synchronous region and oscillatory region at certain LRC probability 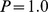 are exposed clearly. And the remarkable enhancement of synchronization transitions induced by LRCs under moderate time delay is also indicated explicitly. From Fig 8 the optimal combinations of time delay and LRC probability on synchronization transitions in delayed Newman-Watts SWNNs are revealed intuitively, which may has a useful impact for actual biological systems.

### The Universality of Time Delay Induced Synchronization Transitions

In order to test the universality of time delay induced synchronization transitions, heterogeneous Newman-Watts SWNNs are considered. Diversity is introduced to system parameter 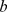, i.e., the values of 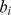 are different in the network. And it satisfies the following Gaussian distribution:
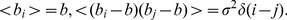
(10)


**Figure 9:**
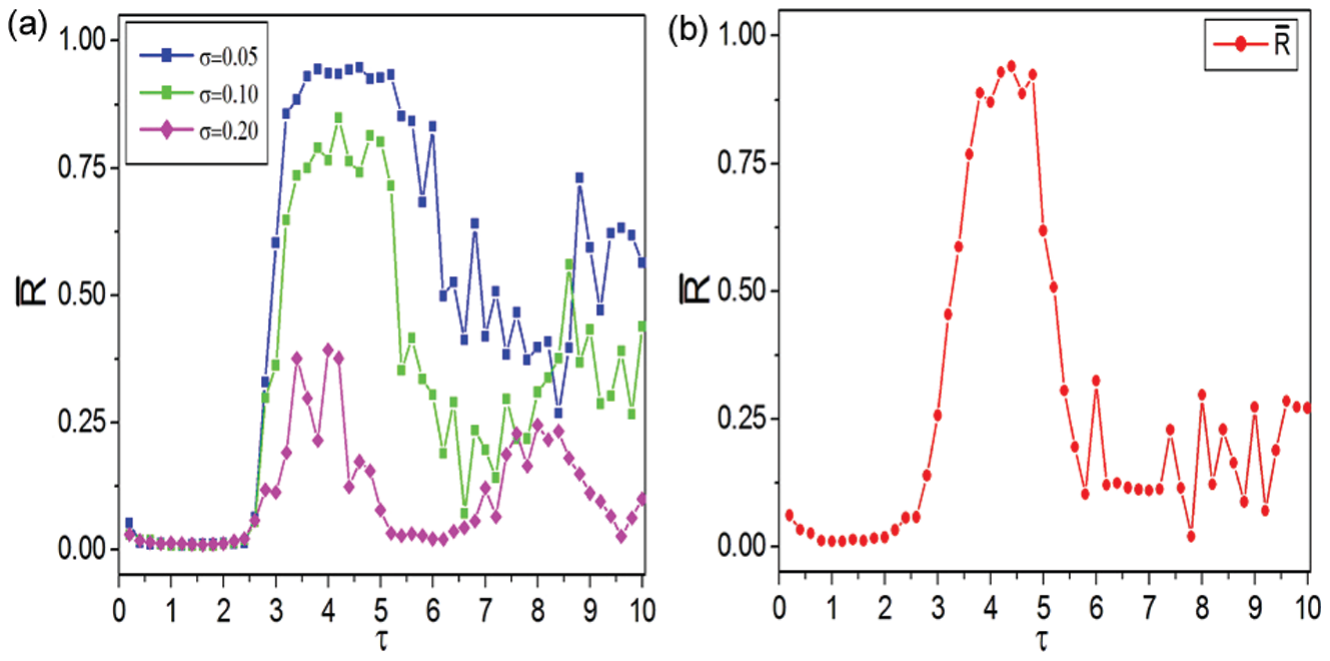
The universality of time delay induced synchronization transitions. (a) Dependence of synchronization parameter 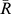 on time delay 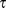 for different diversity 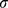. (b) Dependence of synchronization parameter 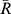 on time delay 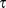 for the new coupling.



The value of 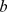 is fixed at 0.07. Here 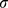 is the standard deviation of the Gaussian probability distribution of system parameter 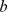. It indicates the strength of the diversity in delayed Newman-Watts SWNNs. Fig. 9(a) shows the dependence of synchronization parameter 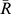 on time delay 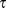 for different diversity 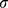 at LRC probability 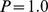. Although synchronization transition becomes less profound as diversity increases, similar time delay induced synchronization transitions can be observed in heterogeneous Newman-Watts SWNNs. More importantly, all these synchronization transitions appear at approximatively same 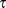. It indicates that time delay plays a significant role in synchronization transitions in Newman-Watts SWNNs. Moreover, to further test the generality of our findings, the following new coupling form is used:
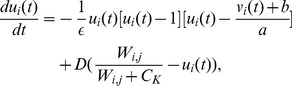
(11)where

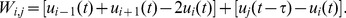
(12)


This type of coupling has been widely used in neural models and excitable complex networks. In simulations, we set 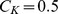. Fig. 9(b) displays the dependence of synchronization parameter 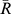 on time delay 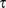. Similar time delay induced synchronization transitions can also be observed for the new coupling form. Now we can conclude that time delay induced synchronization transitions in Newman-Watts SWNNs are a robust phenomenon. The results revealed in present paper are universal.

## Conclusions

In conclusion, time delay and long-range connection induced synchronization transitions in Newman-Watts small-world neuronal networks are systematically investigated by synchronization parameter and space-time plots. We have found four distinct parameter regions, i.e., asynchronous region, transition region, synchronous region and oscillatory region, at certain LRC probability 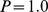 as time delay is increased. Interestingly, desynchronization and oscillating behaviour of the order parameter are observed in oscillatory region. More importantly, the mechanisms of synchronous oscillations and the transition from non-synchronization to complete synchronization are discussed. We consider the spatiotemporal patterns obtained in delayed Newman-Watts SWNNs are the competition results between long-range drivings and neighboring interactions. And our point of view has been verified by LRD proportion, which can also reveal the four distinct parameter regions clearly. In addition, for moderate time delay, the synchronization of neuronal network can be enhanced remarkably by increasing LRC probability. Furthermore, lag synchronization has been found between weak synchronization and complete synchronization as LRC probability 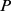 is a little less than 1.0. Finally, the two necessary conditions, moderate time delay and large numbers of LRCs, are exposed explicitly for synchronization in delayed Newman-Watts SWNNs.

As we know that synchronization transitions in neuronal networks are very important issues in related research fields and are associated with some specific physiological functions. A systematical investigation of synchronization transitions induced by time delay and long-range connection is expected to be useful both for theoretical understandings and practical applications. The results obtained in the present paper are universal. Similar time delay induced synchronization transitions can also be observed for heterogeneous Newman-Watts SWNNs and the new coupling form. We do hope that our work will be a useful supplement to the previous contributions and will have a useful impact in related fields.
